# Nephroscope-Assisted Debridement of Pancreatic Necrosis Using the Palanivelu Hydatid Trocar Cannula System: A Novel Technique

**DOI:** 10.7759/cureus.83851

**Published:** 2025-05-10

**Authors:** Bhavana K Satwick, Surendra K Mathur, Roysuneel Patankar

**Affiliations:** 1 Department of Surgery, Zen Hospital, Mumbai, IND; 2 Department of Gastrosurgery, Zen Hospital, Mumbai, IND

**Keywords:** necrosectomy, nephroscopic, palanivelu trocar, pancreatic necrosis, step-up approach

## Abstract

Percutaneous catheter drainage has been proven to be an effective treatment for infected pancreatic necrosis and pancreatic abscess. In cases with failure of drainage of pancreatic necrosis by percutaneous catheters, video-assisted retroperitoneal debridement (VARD) has been recommended. The Palanivelu hydatid trocar cannula system has been developed for its use in laparoscopic management of hydatid cyst disease, as it allows for simultaneous irrigation and suction of daughter cysts. We describe our technique of nephroscopic-assisted drainage of infected pancreatic necrosis using the Palanivelu hydatid trocar cannula system, which, to the best of our knowledge, has not been described in the literature to date.

## Introduction

Acute necrotizing pancreatitis accounts for 10% of cases of acute pancreatitis, and these cases tend to have a higher morbidity and mortality. Percutaneous catheter drainage of necrotic pancreatic collections is indicated in the setting of infected pancreatic necrosis or pancreatic abscess, sepsis, and failure of conservative management [1]. A minimally invasive step-up approach as compared to open necrosectomy is preferred for pancreatic necrosectomy due to reduced incidence of major complications and reduced mortality post-procedure and to avoid a more morbid procedure of open surgical necrosectomy [2, 3]. The Palanivelu hydatid trocar cannula system has been developed for its use in laparoscopic management of hydatid cyst disease, as it allows for simultaneous irrigation and suction of daughter cysts [4]. Here we describe a novel technique of nephroscope-assisted debridement of infected pancreatic necrosis using the Palanivelu hydatid trocar cannula system, which, to the best of our knowledge, has not been described in the literature to date.

## Case presentation

**Figure 1:**
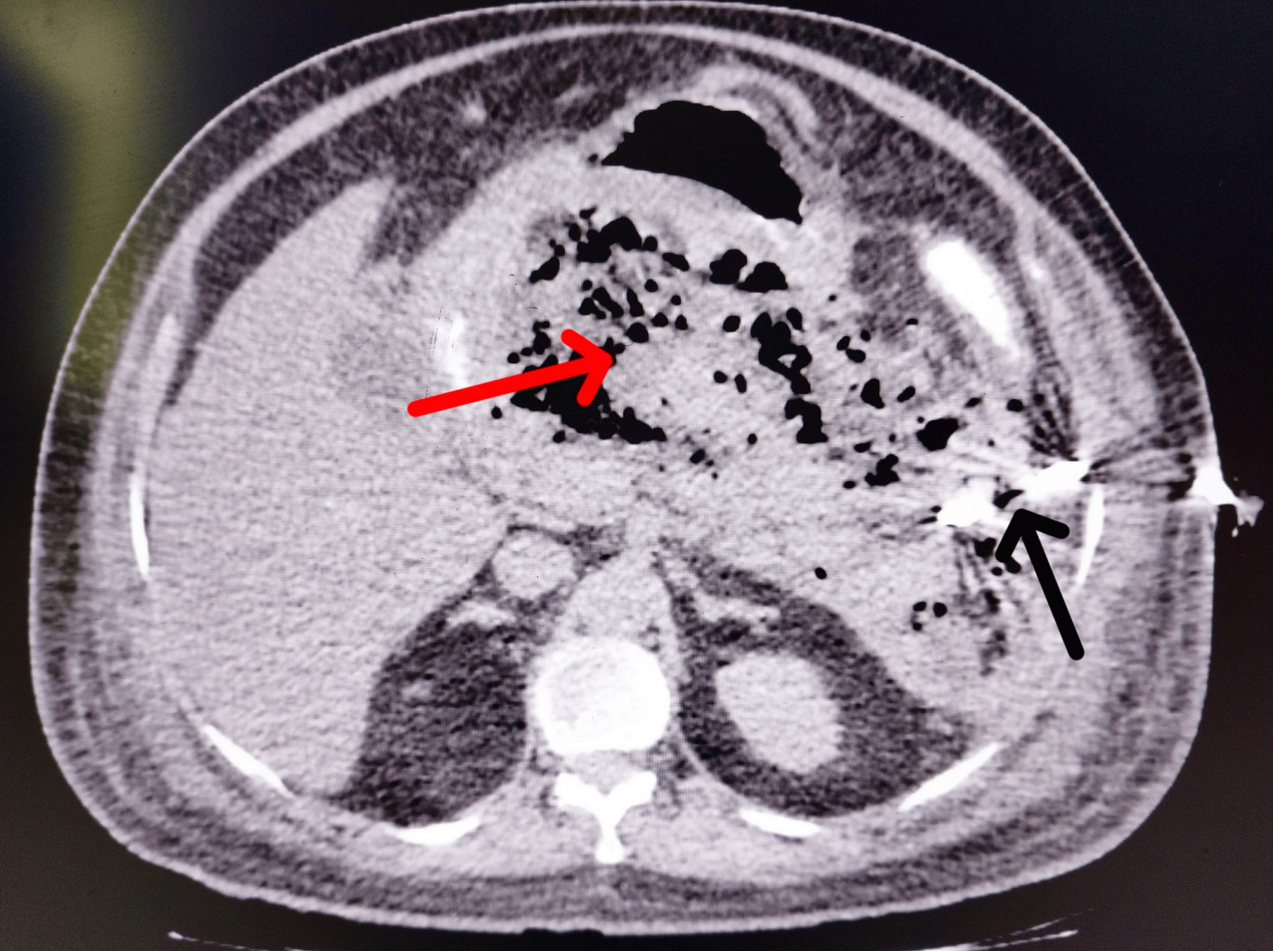
CT scan image showing pancreatic necrosis The red arrow shows a necrotic collection in the region of the head of the pancreas. The black arrow shows a percutaneous drainage catheter draining a necrotic collection in the region of the tail of the pancreas.

A 49-year-old male patient who was diagnosed with necrotizing pancreatitis with necrotic pancreatic collections in the region of the head and tail of the pancreas underwent CT-guided percutaneous catheter placement in the necrotic collection in the region of the tail of the pancreas through the left side of the abdomen at another hospital. The patient presented to us three weeks later with a percutaneous drainage catheter in situ. A repeat CT scan was done, which showed an 18x17x13 cm necrotic collection in the region of the head of the pancreas and another smaller necrotic collection in the region of the tail of the pancreas with no communication between the two cavities (Figure 1).

Due to the failure of conservative management, a decision was made for CT-guided percutaneous drainage of the necrotic collection in the region of the head of the pancreas through a separate drainage catheter. A 12 Fr percutaneous drainage catheter was placed through the anterior abdominal wall into the collection in the region of the head of the pancreas under CT guidance. A nasojejunal tube was inserted to maintain enteral nutrition. As the condition of the patient failed to improve even after percutaneous drainage, a repeat CT scan done one week later showed persistence of necrotic collection with no significant change in its size. Hence, the decision was taken to use a step-up minimally invasive approach using a nephroscope for pancreatic necrosectomy.

Our surgical technique

**Figure 2:**
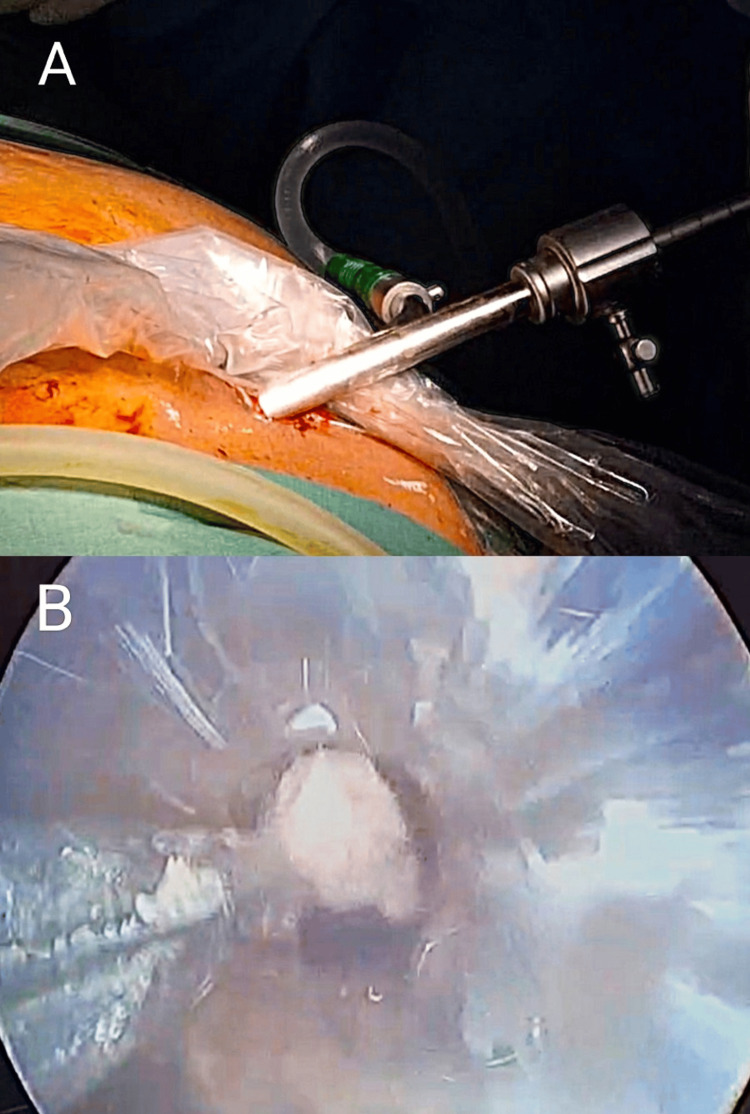
Intraoperative image of nephroscopic-assisted drainage of pancreatic necrosis using the Palanivelu hydatid trocar cannula system Adherent necrotic tissue was grasped with stone-holding forceps under nephroscopic vision (A) and excised (B).

The patient was placed in a supine position. The previously placed percutaneous drainage catheter opening in the left anterior axillary line draining the collection in the region of the tail of the pancreas was widened, and the collection was drained, followed by placement of an abdominal drain in the cavity in the region of the tail of the pancreas. For drainage of the larger necrotic collection in the region of the head of the pancreas, the previously placed percutaneous drainage catheter opening was widened. A guide wire was passed through the percutaneous drainage catheter under fluoroscopic guidance, and the catheter was removed. Serial dilatation of the drainage tract was done with Amplatz renal dilators. The Palanivelu hydatid trocar cannula system with side suction channel was placed through the dilated drainage tract. A 24 Fr nephroscope was passed through the Palanivelu hydatid trocar, and suction was connected to the suction side channel of the system. This allowed us to irrigate the cavity and simultaneously aspirate any loose necrosome through the suction channel of the system. Parts of the necrotic tissue that were adherent were grasped with stone-grasping forceps under nephroscopic vision and removed (Figure 2).

**Figure 3:**
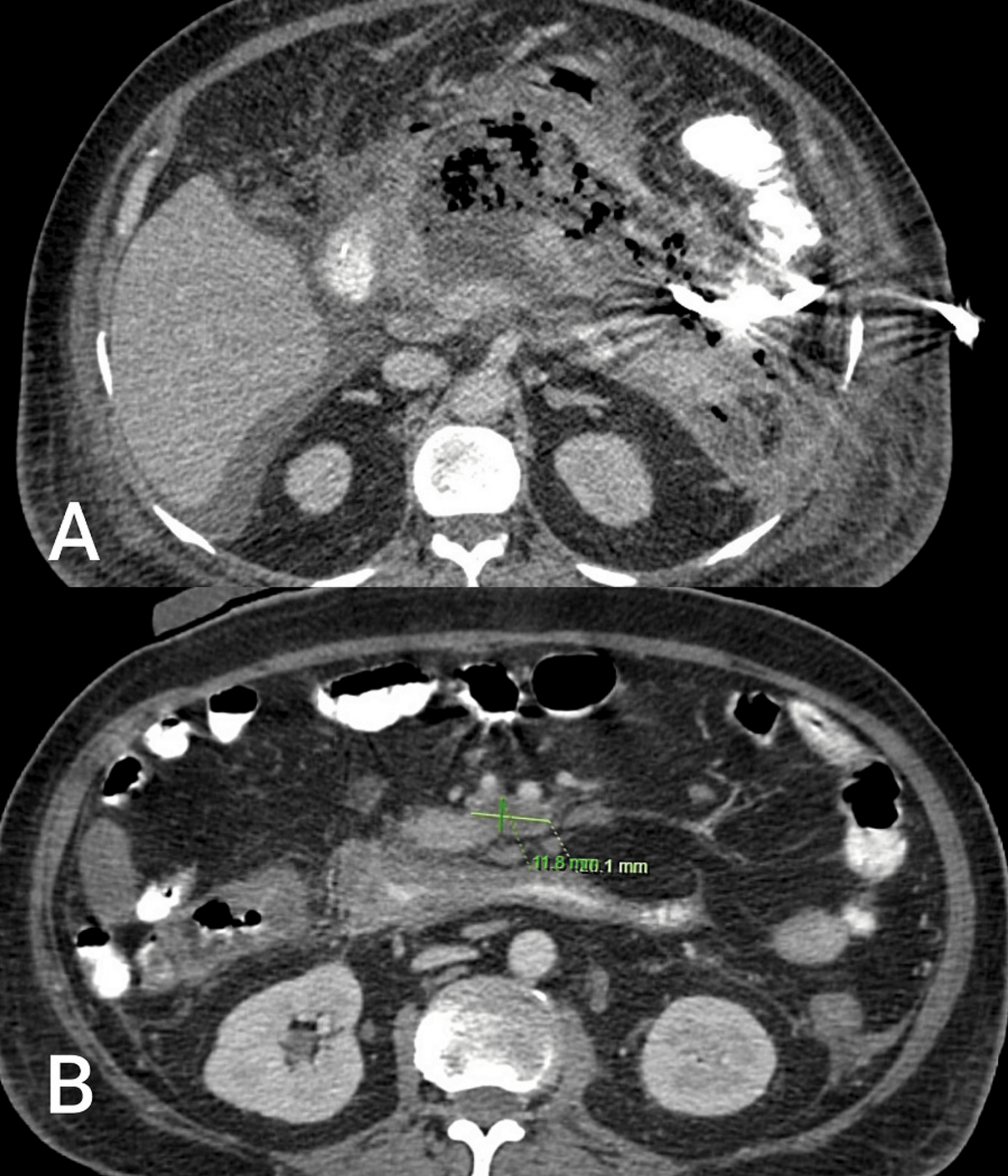
CT scan images of necrotic pancreatic collection (A) Before nephroscopic-assisted debridement; (B) After nephroscopic-assisted debridement

The necrotic cavity was thoroughly washed with normal saline until the returning fluid was clear. Around 400 ml of necrotic material was drained intraoperatively. Two drains were placed in this cavity in the region of the head of the pancreas for closed saline lavage in the postoperative period. Postoperative recovery was uneventful. Saline lavage of the necrotic cavity was done in the postoperative period till the returning fluid was clear, with a daily drain output of 100-150 ml, which reduced gradually over a period of time. The nasojejunal feeding tube was removed on postoperative day 15. The patient was discharged on postoperative day 15 with abdominal drains in situ. The patient was followed up for a period of six weeks with monitoring of daily drain output, which had reduced significantly. A check CT scan done six weeks later showed a reduction in necrotic collection; hence, drains were removed. A comparison of the size of the necrotic collection before nephroscope-assisted debridement and six weeks after the procedure is shown in the image below (Figure 3). 

## Discussion

**Figure 4:**
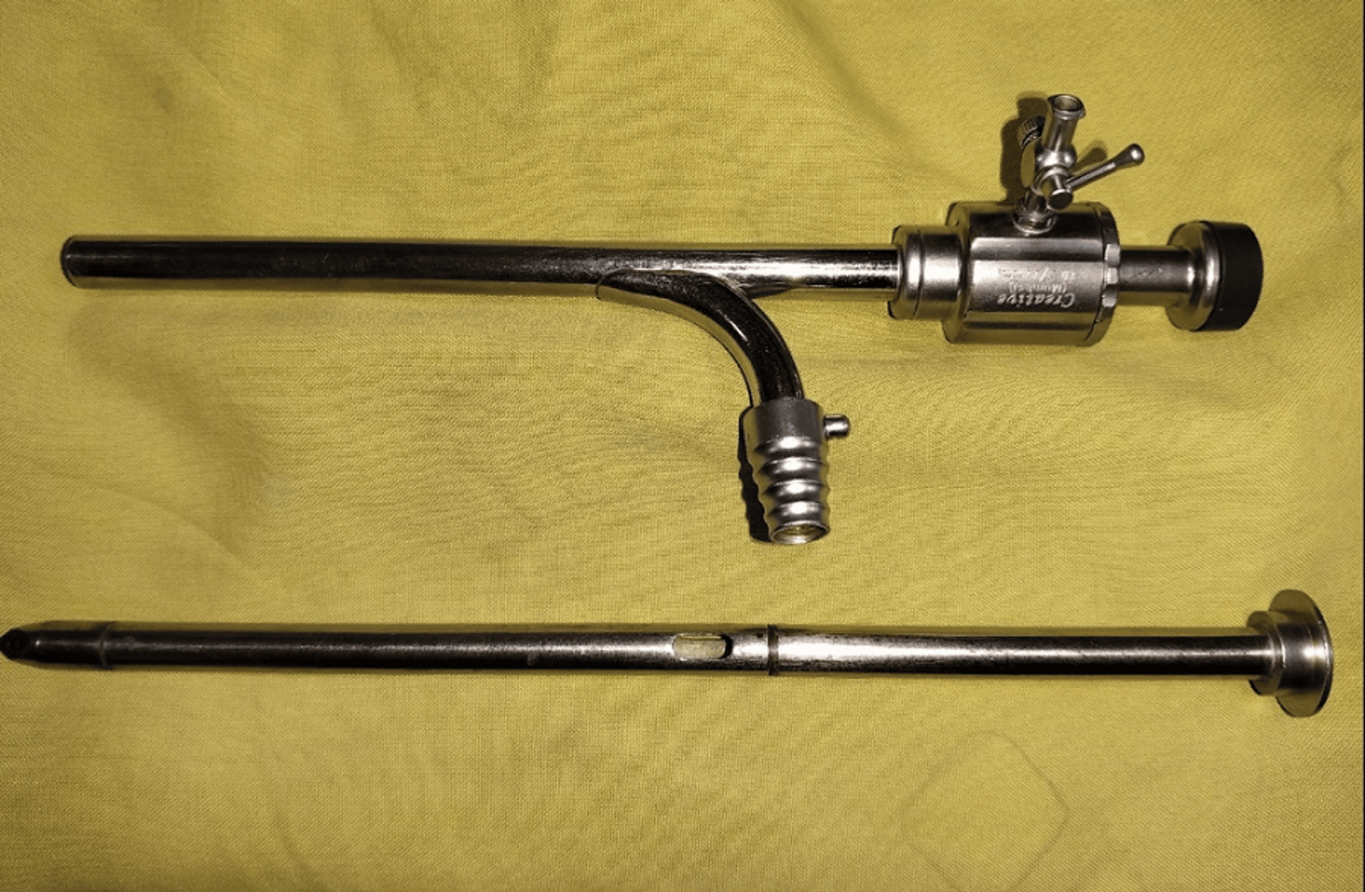
The Palanivelu hydatid trocar cannula system with a wide-bore side suction channel

Pancreatic necrosis is defined as diffuse or focal areas of nonviable pancreatic parenchyma involving >3 cm in size or >30% of the pancreas. Walled-off pancreatic necrosis is a well-recognized complication of necrotizing pancreatitis. Intervention is not required for asymptomatic pancreatic or extra-pancreatic necrosis regardless of size, location, and extension. Surgical intervention is required in cases of failure of conservative management, sepsis, or collections causing luminal obstruction. In stable patients with infected necrosis, radiologic or surgical drainage should be delayed, preferably for four weeks, to allow liquefaction of the contents and development of a fibrous wall around the necrosis. In symptomatic patients with infected necrosis, a minimally invasive step-up approach is preferred over open necrosectomy due to lesser morbidity and mortality [3, 5]. Video-assisted retroperitoneal debridement (VARD) is the minimally invasive surgical technique currently practiced for pancreatic necrosis [6]. VARD involves accessing the necrotic cavity with a small incision and removing the pancreatic necrosis initially under direct vision, followed by further debridement under videoscopic assistance [7]. While adhering to the principles of minimally invasive necrosectomy, we performed necrosectomy with nephroscopic assistance in our patient. The Palanivelu hydatid trocar cannula system is classically designed for laparoscopic management of hydatid cyst disease (Figure 4).

The trocar is 29 cm long and hollow throughout its length to accommodate a suction cannula. The cannula is 26 cm long with an inner diameter of 12 mm. It has two channels, one for gas insufflation and another for suction. The suction channel has an inner diameter of 10 mm, and its outer nozzle is designed such that the suction tube can fit onto it in an airtight manner. When used in hydatid cyst disease, this system allows irrigation of the daughter cysts along with simultaneous suction of the daughter cysts via the suction channel [4]. The same principles were applied when using this system in pancreatic necrosectomy. The wide inner diameter of the main channel allowed us to irrigate the necrosis with simultaneous suction via the 10 mm suction channel. The use of a 24 Fr nephroscope allowed us to directly visualize necrotic tissue during irrigation. For the necrosis that was adherent and could not be removed by irrigation alone, we used stone-grasping forceps and debrided the tissue under nephroscopic vision. In our patient, we could achieve adequate necrosectomy via this novel technique with similar postoperative outcomes as compared to VARD.

## Conclusions

In cases of pancreatic necrosis, surgical intervention is required in cases of failure of conservative management. In our patient, nephroscope-assisted drainage and necrosectomy using the Palanivelu hydatid trocar cannula system were done, as there was a failure of clinical improvement on conservative management and even after percutaneous catheter drainage. The Palanivelu hydatid trocar cannula system is designed specifically for its use in laparoscopic management of hydatid cyst disease, and its use in pancreatic necrosectomy has not been described in the literature to date. Here we have demonstrated that nephroscope-assisted debridement of pancreatic necrosis using the Palanivelu hydatid trocar cannula system is a novel and effective minimally invasive method of pancreatic necrosectomy; however, further large-scale studies using this technique are recommended.
